# The Burden of HPV-Related Hospitalizations: Analysis of Hospital Discharge Records from the Years 2015–2021 from a Southern Italian Region

**DOI:** 10.3390/pathogens12050725

**Published:** 2023-05-17

**Authors:** Giuseppe Di Martino, Fabrizio Cedrone, Pamela Di Giovanni, Livia Tognaccini, Edoardo Trebbi, Ferdinando Romano, Tommaso Staniscia

**Affiliations:** 1 Department of Medicine and Ageing Sciences, “G. d’Annunzio” University of Chieti-Pescara, 66100 Chieti, Italy; 2 Unit of Hygiene, Epidemiology and Public Health, Local Health Authority of Pescara, 65100 Pescara, Italy; 3 Hospital Management, Local Health Authority of Pescara, 65100 Pescara, Italy; cedronefab@gmail.com; 4 Department of Pharmacy, “G. d’Annunzio” University of Chieti-Pescara, 66100 Chieti, Italy; 5 School of Public Health, “La Sapienza” University of Rome, 00100 Rome, Italy; 6 Department of Public Health and Infectious Diseases, “La Sapienza” University of Rome, 00100 Rome, Italy; ferdinando.romano@uniroma1.it

**Keywords:** HPV, epidemiology, Italy, hospital admissions, cancer, prevention

## Abstract

(1) Background: The human papillomavirus (HPV) is the most common agent related to sexually transmitted infections in the general population. Its genotypes are classified into two main classes, high-risk genotypes and low-risk genotypes, according to their capacity to induce cancers. The low-risk class (types 6 and 11) is associated with anogenital and genital lesions. The high-risk class is responsible for up to 4.5% of all new cancer cases yearly. The aim of this study was to evaluate the incidence of HPV-related hospitalizations and its trend in a southern Italian region for the years 2015–2021. (2) Methods: This was a retrospective study performed in the Abruzzo region, Italy. All admissions for the period 2015–2021 were extracted from the hospital discharge record (HDR). (3) Results: During the study period (2015–2021), a total of 5492 hospitalizations attributable to HPV infection occurred in the Abruzzo region, Italy. A significant number of admissions were related to cervical cancer (3386 cases) and genital warts (638 cases). The trend declined for all diagnoses except for penile cancer admissions. Considering the first year of the pandemic (year 2020), a decrease in the standardized incidence of the majority of the diseases considered was reported, particularly among cases of cervical cancer. (4) Conclusions: HPV-related hospitalizations decreased in Abruzzo during study period. These results could be useful to LHAs and policy-makers in improving vaccination coverage and screening adherence.

## 1. Introduction

The human papillomavirus (HPV) is the most common agent related to sexually transmitted infections in the general population [1,2].

The number of HPV genotypes that have been identified is almost 200 [3,4], and the epidemiology of HPV varies across countries [4].

HPV genotypes are classified into two main classes, high-risk genotypes and low-risk genotypes, according to their capacity to induce cancers. The low-risk class, mainly represented by types 6 and 11, is associated with anogenital warts and low-grade genital lesions [5]. On the other hand, the high-risk class (mainly HPV types 16 and 18) is responsible for up to 4.5% of all new cancer cases yearly. In particular, it is responsible for cervical cancers [6], anal cancers, penile cancers, and over 31% of oropharyngeal cancers [7]. Considering that cervical cancer is actually the fourth most common female cancer [8], particularly among women aged between 35 and 44 years [9], with over 300,000 yearly deaths estimated, it represents a heavy burden for healthcare services. In addition, other HPV-related conditions showed a substantial burden of disease across highly developed countries, with trends varying by country [10,11,12]. Genital warts represent another HPV-related condition that mainly affects younger patients (mainly caused by genotypes 6 and 11), with an annual hospitalization rate of over 8/100,000 inhabitants [13]. Other cancers are also associated with HPV infections. In particular, anal cancers were strongly associated with HPV infection, with surges of near 90% of cases in European countries [8,10]. The most important risk factors for anal cancers other than HPV are men who have sex with men and HIV infections. [10]. Additionally, female genital tract cancers and oropharyngeal cancers were also linked to HPV infection. In particular, the incidences of head and neck cancers strongly increased worldwide and are suspected to overcome cervical cancer in some developed countries during the next decade [7]. Particularly in North America, the fraction of oropharyngeal cancers attributable to HPV is higher than 60% compared to some European countries, which reported an attributable fraction lower than 40% [7]. These differences could be explained by different behaviors across countries.

Although Pap tests and HPV-DNA screening tests are able to early detect cervical cancers, there is a lack of screening tools to prevent other types of HPV-related cancers. Therefore, the most important strategy for facing off against HPV-related diseases is vaccination [3]. A safe and effective 9-valent HPV vaccine is available that protects against all the high-risk HPV genotypes involved in cancer development [14].

The World Health Organization aims to eradicate cervical cancer by 2030 if nearly 90% of female adolescents are vaccinated against HPV [15]. To meet this challenge, since 2009, the Italian Ministry of Health has offered HPV vaccination to all females aged over 12 years. Since the 2005 birth cohort, the vaccination campaign has been extended to male subjects [16]. In fact, a recent study demonstrated a strong impact of HPV vaccination on types of diseases other than cervical cancer. Recently, the estimation of data in Italy was presented in several studies [17,18,19,20] trying to evaluate the burden of HPV diseases and their related hospitalizations; however, no epidemiological studies have been covered most recent years. The pandemic heavily impacted health services with high hospitalization rates for COVID-19, forcing the modification of several hospitals into COVID-19 centers. Although this situation helped healthcare services to fight the pandemic, it had indirect consequences on the care for other diseases. Regarding this point, no studies evaluated the impact of the COVID-19 pandemic on HPV-related hospitalizations. To this extent, the aim of this study was to evaluate the incidence of HPV-related hospitalizations and its trend in a southern Italian region for the years 2015–2021.

## 2. Materials and Methods

### 2.1. Study Design and Setting

This was a retrospective study performed in the Abruzzo region, Italy. Abruzzo is a southern Italian region that counts over 1.3 million inhabitants and whose healthcare services are organized into four local health authorities (LHAs) [21]. Data related to hospital admissions were extracted from the hospital discharge record (HDR) of the Abruzzo region, taking into account all admissions performed during the years 2015–2021 and also including those performed in other Italian regions by inhabitants from Abruzzo. The HDR included data on patient demographics, a diagnosis-related group (DRG) code used to classify the admission, up to six diagnoses (one principal diagnosis and up to five comorbidities), and up to six possible procedures that occurred during the hospitalization. The diagnoses and procedures were coded according to the International Classification of Disease, 9th Revision, Clinical Modification (ICD-9-CM), the National Center for Health Statistics (NCHS), and the Centers for Medicare and Medicaid Services External, Atlanta, GA, USA [22].

### 2.2. Outcomes of Interests and Inclusion Criteria

In order to identify all HPV-related hospitalizations, the following codes were considered, according to the ICD-9-CM [23] and prior studies [24]:

- Condyloma acuminatum (078.11);
- Head and neck cancers (140.0–149.9, 195.0, 230.0 and 235.1);
- Anal cancers (154.2–154.8, 230.5–230.6);
- Cervical cancers (180.0–180.9, 233.1, 622.1, 654.6 and 795.0–795.1);
- Cancers of the vagina, labia, and clitoris (184.0–184.8);
- Penile cancers (187.1–187.9, 233.5).
- Among procedures, the following codes were also considered:
- Cervix conization (67.2);
- Cervical lesion cauterization (67.32);
- Cervical lesion cryosurgery (67.33).

If a patient experienced multiple admissions for the same diseases, it was counted only for the first time. Codes 233.3 (carcinoma in situ of other and unspecified female genital organs) and 184.9 (malignant neoplasm of female genital organ, site unspecified) were referred to lesions in an unspecified site and, in order to avoid the overestimation of the incidence of HPV-related hospitalization, they were not included.

### 2.3. Statistical Analysis

Annual and total hospitalization rates for each HPV-related disease (cervical cancer; vulval and vaginal cancer; penile cancer; oropharyngeal cancer; anal cancer; and genital warts) were calculated per 100,000 inhabitants using the related attributable fractions, according to most recent literature [13,16]: cervical cancer, 100%; genital warts, 100%; anal cancer, 88%; genital tract cancer, 78%; penile cancer, 51%; oropharyngeal cancer, 24%. Hospitalization rates were standardized for age and gender according to the Abruzzo population in the first year of the study. The Joinpoint model (Joinpoint version 4.6.0.0, 2018) was used to evaluate the time trends of the standardized rates and the average annual percent change (APC). The APC must be considered a summary measure of the trend over time interval that is computed as a weighted average of the annual percent change. The final model was based on linear segments connected at joinpoints that represent the best fit of the observed data. The best model was evaluated by the permutation test. For all tests, a *p*-value less than 0.05 was considered significant. The statistical analysis was performed with STATA v14.2 software (StataCorp LLC, College Station, TX, USA).

## 3. Results

During the study period (2015–2021), a total of 5493 hospitalizations attributable to HPV infection occurred in the Abruzzo region, Italy. The most frequent admissions were related to cervical cancer (3386 cases) and genital warts (638 cases), as reported in Table 1. Among cervical cancer patients, the greatest number of admissions occurred for the class of patients aged 15–64 years (49.6%). Among patients with penile cancer, on the other hand, the most represented class was the age range of 65–80 years (43.8%). Regarding genital warts, the disease was equally distributed across gender (52% among female), and the most represented age class was 15–64 years (404 cases, 63.3%).

Table 2 shows trends in HPV-related hospitalizations across the study period by disease. In particular, the trend declined for all diagnoses except for penile cancer admissions, which grew from 1.9 admissions/100,000 in 2015 to 2.3 admissions/100,000 in 2021. Among analyzed conditions other than penile cancer, a great reduction was reported among genital warts, the incidence of which decreased from 8.5 admissions/100,000 in 2015 to 5.9 admissions/100,000 in 2021. Oropharyngeal cancers and vulvar/clitoral cancers remained stable across study period. oropharyngeal cancers ranged between 5.5/100,000 in 2015 to 5.2/100,000 in 2021, and vulvar/clitoral cancers ranged between 2.6/100,000 in 2015 to 2.5/100,000 in 2021.

**Figure 1:**
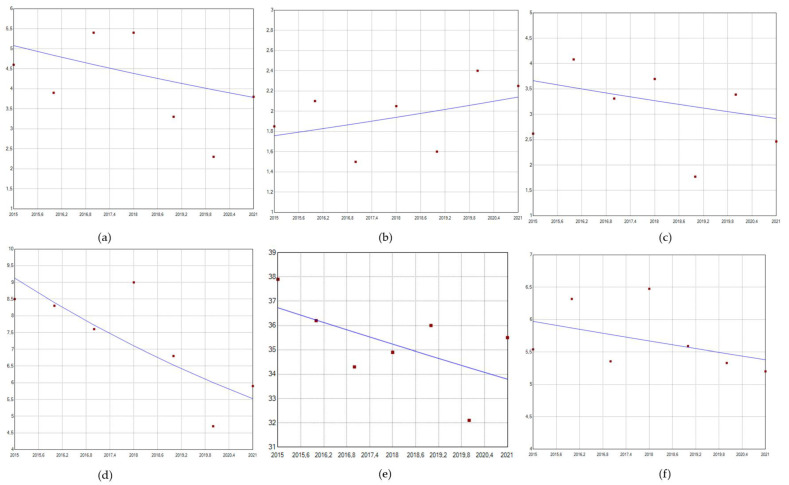
Trends in HPV-related admission (hospitalization rate/100,000 population). (**a**) Anal cancer; (**b**) penile cancer; (**c**) genital-tract cancer; (**d**) genital warts; (**e**) cervical cancer; (**f**) oropharyngeal cancer.

Figure 1 shows that admissions for genital warts significantly decreased over the study period, with an APC of −8.03 (95%CI: −11.91–−0.33). The other most important reductions refer to anal cancer (APC −4.79, 95%CI −16.91–6.43) and female genital cancers (APC −3.72, 95%CI −11.21–7.20). An increasing trend was reported for penile cancer over the study period (APC 3.30, 95%CI −5.10–12.50).

**Figure 2:**
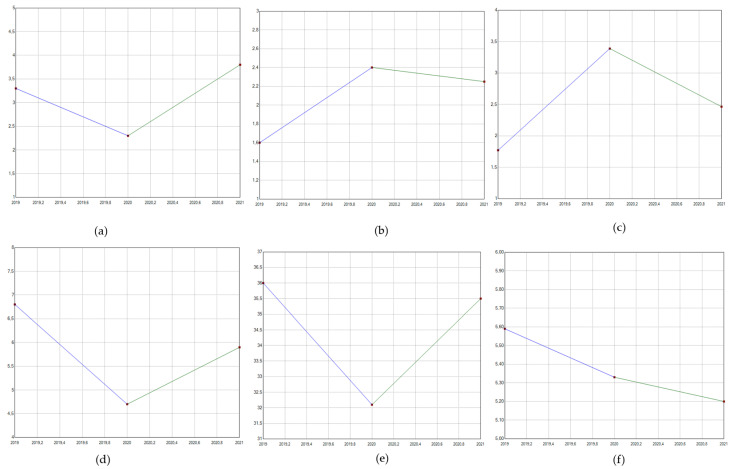
Trends in HPV-related admission (hospitalization rate/100,000 population) during pandemic years. (**a**). Anal cancer; (**b**) penile cancer; (**c**) genital-tract cancer; (**d**) genital warts; (**e**) cervical cancer; (**f**) oropharyngeal cancer.

Considering the first year of the COVID-19 pandemic (year 2020), a decrease in the standardized incidence of a large part of the diseases considered was reported, particularly among cervical cancer (PC −10.8 from 2019 to 2020), genital warts (PC −30.9 from 2019 to 2020), oropharyngeal cancer (PC −4.7 from 2019 to 2020), and anal cancer (PC −30.3 from 2019 to 2020). On the other hand, a strong increase was reported for all these conditions in the year 2021. Only penile cancer and genito-urinary tract cancers reported an increase between the years 2019 and 2020, with PCs of 50.0 and 91.3, respectively. Oropharyngeal cancer admissions also decreased in the years 2020 and 2021, as reported in Figure 2.

Among diseases concerning both genders, the highest incidence was observed for oropharyngeal cancers, particularly among older classes of male subjects. Gender differences in the incidence of anal cancer were observed during first two years of the study, but this difference was reduced during study period, as reported in Table 3.

Oropharyngeal cancer showed a higher incidence among older men (from 1.3 to 1.6/1000 inhabitants) compared to women (from 0.6 to 0.5/1000 inhabitants).

## 4. Discussion

The present study reported the trends of hospitalization for HPV-related diseases from 2015 to 2021 in the Abruzzo region, Italy. Cervical cancer demonstrated the highest incidence rate, with a yearly average rate of 36.6 cases/100,000. Despite this, the hospitalization rate appears lower in the Abruzzo region compared to other Italian regions and to Italian data overall [13,16]. Overall, the decreasing trends for all diseases confirm the results from similar studies performed in Italy [13,20,24]. Only the admission rate for penile cancer demonstrated an increasing trend, although it was not significant across the study period. Surely, the incidence of penile cancer is increasing in many countries in recent years [25] and, despite the lack of significant results, it can heavily impact the healthcare system in the Abruzzo region, and a preventive strategy should be implemented. The results of this study highlight how the greatest decreases in admission rates were reported for genital warts, cervical cancer, and anal cancer. The most important factor associated with this decrease was probably the implementation of the HPV vaccination campaign, as suggested by the previous literature. In particular, a recent systematic review [26] that pooled data from 14 high-income countries highlighted the reduction in genital wart and CIN2+ lesion diagnoses due to HPV vaccination. In addition, these results are in line with similar studies performed in Italy during the last decade [13,17,27,28,29,30,31].

Despite these results, Abruzzo has low vaccination coverage against HPV compared to the threshold required by the Italian Ministry of Health [32,33]. It is sure that the impact of the HPV vaccination campaign will show its most important results in the next few years, according to the growing vaccination rate and thanks to the extension of the free vaccination to young males from the 2006 cohort, which began in Italy in 2017–2019 [12]. This can help also in filling the gap in differences between genders for anal cancers. In addition, cervical cancer screening has an important impact on cancer admissions. The screening uptake varies across Italian regions, and the Abruzzo region reports an adherence rate of 78% (years 2017–2019), which is in line with the national mean [26]. The expected decrease in screening uptake that occurred during the pandemic years will surely impact cancer care in the next years, with an increase in the number of avoidable cancer deaths [34]. Regarding oropharyngeal cancers, the results from this study are in line with the previous literature in which reports of the admission rate for HPV-related head and neck cancers ranged between 5.6 and 33/100,000 [26]. The unchanged trend among head and neck cancers could have a possible reason: compared to cervical cancer, whose reduction resulted from routine screening gynecological evaluations and HPV vaccination that was initiated primarily among women, the responsibility for the diagnosis of oropharyngeal cancer is generally not assigned to a medical specialty or a routine screening procedure. This situation frequently results in a late diagnosis that occurs when the patient becomes symptomatic [27]. In addition, several other risk factors are known to be linked to head and neck cancers, such as having a smoking habit, drinking alcohol, and other environmental factors that are strongly associated with oropharyngeal cancers. The gender difference was particularly highlighted in the incidence of oropharyngeal cancer and is probably linked to different risk factors relating to life exposure among men.

Penile cancer was the only affliction that was reported to have an increasing trend, despite the fact that the reported incidence rates remained lower than nation data [24]. The greatest problem with penile cancer was linked to the lack of a specific screening program and to its multiple pathogenic pathways. In particular, although one-half of penile cancer was linked to HPV, a great part was associated with chronic inflammation. Circumcision in childhood significantly reduces the prevalence of the condition, and this is one of the main reasons for epidemiological differences across countries [35]. In addition, the introduction of the HPV vaccination among men in Italy was too recent, and its impact cannot be actually evaluated.

Regarding the pandemic period, COVID-19 heavily impacted healthcare systems, causing a reduction in hospital admissions for many diseases [36]. Data reported for HPV-related hospitalizations during the year 2020 appear to confirm this general trend, with a global reduction in hospital admissions for all diseases except for penile cancer. The observed reduction in admissions could be explained by the adaptation of the healthcare system to the pandemic response: healthcare services focused their attention on SARS-CoV-2 patients, and the conversion of great proportions of surgical units into COVID wards was needed. This situation strongly affected routine surgical activity, causing a decrease in admissions for all oncological diseases [37]. The first pandemic year showed a strong decrease, particularly for genital warts admissions, which showed the most important reduction (Figure 2). In parallel to the decrease in all surgical activities, all non-urgent procedures, such as the treatment of genital warts, were postponed for longer compared to oncological surgery procedures. It should be also noted that the treatment for genital warts frequently requires only ambulatory care, and the evaluation of its incidence can be underestimated with HDR.

Finally, the overall decrease in admission trends for all included diagnosis confirmed the national trend [17] in most recent years, mainly due to screening programs and the improvement in vaccination coverage.

Overall, the results reported by this study were similar to national data recently reported by Restivo et al. [24]. The decreasing trend for all diseases in the Abruzzo region except for penile cancer confirmed the national trend. Regarding penile cancers, despite the non-significant increases reported, the incidence of penile cancers in the Abruzzo region remains lower compared to the Italian result [24]. Despite the lack of data on screening adherence and vaccination information from admitted patients, these results show the strong impact of preventive measures on HPV-related hospitalizations. The novelty apported by this study was the particular focus on pandemic period. In fact, cancelled or deferred admission due to pandemic must be recovered during next years, and healthcare services need to handle the overload, particularly for oncological diseases. In particular, the deferral for the hospitalization of time-sensitive diseases such as cancers can lead to the worsening of cancer staging at surgery and to preventable death. At the same time, the pandemic strongly impacted cancer screening, with a decrease of almost 50% in cervical cancer screenings [27]. Effective interventions are also required to improve the capacity of screening services to handle the pandemic decrease.

### Strength and Limitations

The strength of this study is the large sample analyzed and the long study period considered. This is the first study to have been conducted in the Abruzzo region considering all the hospital admissions related to HPV. In addition, this the first study conducted in Italy that evaluates the most recent years (2019–2021).

However, the results of this study should be considered in the light of the following limitations. Firstly, the reported diagnoses in HDR were based on ICD-9-CM codes that did not consider the severity of the condition and the HPV genotype. Secondly, in HDR, some diagnostic codes could have been under-reported or incorrectly reported, causing underestimates of the rate. Thirdly, these results were referred to a small Italian region. Finally, this study evaluated only hospital admissions, and it could not consider procedures performed by private practitioners or ambulatory care facilities, especially for genital warts. Thus, this study cannot be used to evaluate the real incidence of HPV-related illnesses.

## 5. Conclusions

HPV-related hospitalizations decreased in Abruzzo during the study period. These results could be useful to LHAs and policy-makers in improving vaccination coverage and screening adherence. A decreasing trend in hospitalizations due to the extension of HPV vaccination to male subjects is expected in the coming years. In parallel, trends should also be analyzed in the future to evaluate the impact of the COVID-19 pandemic on the decrease in vaccination and screening adherence.

## Figures and Tables

**Table 1:** Admission for HPV-related diseases according to age and gender.

Diagnosis	HPV-Attributable Fraction	Gender	Age Group	n	%	Total Admissions	HPV-Related Admissions
Cervical cancer	100%					3386	3386
		Female	0–4	0	0		
			5–14	0	0		
			15–64	1681	49.6		
			65–80	1399	41.3		
			>80	306	9.1		
Penile cancer	51%					361	184
		Male	0–4	0	0.0		
			5–14	0	0.0		
			15–64	88	24.3		
			65–80	158	43.8		
			>80	115	31.9		
Genital tract cancer	78%					365	284
		Female	0–4	0	0.0		
			5–14	0	0.0		
			15–64	82	22.5		
			65–80	183	50.1		
			>80	100	27.4		
Anal cancer	88%					440	387
		Female	0–4	0	0.0		
			5–14	3	0.7		
			15–64	64	14.5		
			65–80	105	23.9		
			>80	53	12.0		
		Male	0–4	0	0.0		
			5–14	5	1.1		
			15–64	78	17.8		
			65–80	77	17.5		
			>80	55	12.5		
Oropharyngeal cancer	24%					2385	572
		Female	0–4	4	0.2		
			5–14	76	3.2		
			15–64	597	25.0		
			65–80	619	26.0		
			>80	287	12.0		
		Male	0–4	6	0.3		
			5–14	67	2.8		
			15–64	268	11.2		
			65–80	234	9.8		
			>80	227	9.5		
Genital warts	100%					638	638
		Female	0–4	0	0.0		
			5–14	11	1.7		
			15–64	204	32.0		
			65–80	75	11.8		
			>80	42	6.6		
		Male	0-4	0	0.0		
			5–14	7	1.1		
			15–64	200	31.3		
			65–80	78	12.2		
			>80	21	3.3		

**Table 2:** Standardized admission rates (/100,000 inhabitants) for HPV-related diseases.

	2015 (SD)	2016 (SD)	2017 (SD)	2018 (SD)	2019 (SD)	2020 (SD)	2021 (SD)
Genital Warts	8.5 (0.6)	8.3 (0.6)	7.6 (0.6)	9 (0.6)	6.8 (0.6)	4.7 (0.5)	5.9 (0.6)
Oropharyngeal cancer	5.5 (0.3)	6.3 (0.3)	5.4 (0.3)	6.4 (0.3)	5.6 (0.3)	5.3 (0.3)	5.2 (0.3)
Anal cancer	4.6 (0.3)	3.9 (0.3)	5.4 (0.3)	5.4 (0.3)	3.3 (0.2)	2.3 (0.2)	3.8 (0.3)
Cervical cancer	37.9 (1.4)	36.2 (1.4)	34.3 (1.4)	44.9 (1.4)	36 (1.4)	32.1 (1.4)	35.5 (1.4)
Genital tract cancer	2.6 (0.2)	4.1 (0.3)	3.3 (0.3)	3.7 (0.3)	1.7 (0.2)	3.4 (0.3)	2.5 (0.2)
Penile cancer	1.9 (0.2)	2.1 (0.2)	1.5 (0.1)	2.1 (0.2)	1.6 (0.1)	2.4 (0.2)	2.3 (0.2)

**Table 3:** Admission rates (/1000 inhabitants) for HPV-related diseases concerning both genders.

Diseases	Gender	Age Class	2015 (SD)	2016 (SD)	2017 (SD)	2018 (SD)	2019 (SD)	2020 (SD)	2021 (SD)
Genital warts									
	Female	15–64	0.2 (0.01)	0.2 (0.01)	0.1 (0.01)	0.1 (0.01)	0.1 (0.01)	0.1 (0.01)	0.1 (0.01)
		65–80	0.1 (0.01)	0.1 (0.01)	0.1 (0.01)	0.1 (0.01)	0.1 (0.01)	0.1 (0.01)	0.1 (0.01)
		>80	0	0	0	0	0.1 (0.01)	0	0
	Male	15–64	0.1 (0.01)	0.2 (0.01)	0.1 (0.01)	0.2 (0.01)	0.1 (0.01)	0.1 (0.01)	0.1 (0.01)
		65–80	0.1 (0.01)	0.1 (0.01)	0.1 (0.01)	0.1 (0.01)	0.2 (0.01)	0.1 (0.01)	0.1 (0.01)
		>80	0	0	0	0	0.3 (0.01)	0	0
Anal cancer									
	Female	15–64	0.1 (0.01)	0.1 (0.01)	0.1 (0.01)	0.1 (0.01)	0.1 (0.01)	0.1 (0.01)	0.1 (0.01)
		65–80	0.1 (0.01)	0.1 (0.01)	0.1 (0.01)	0.2 (0.01)	0.1 (0.01)	0.1 (0.01)	0.1 (0.01)
		>80	0.1 (0.01)	0.1 (0.01)	0.2 (0.01)	0.2 (0.01)	0.1 (0.01)	0.1 (0.01)	0.1 (0.01)
	Male	15–64	0.1 (0.01)	0.1 (0.01)	0.1 (0.01)	0.1 (0.01)	0.1 (0.01)	0.1 (0.01)	0.1 (0.01)
		65–80	0.2 (0.01)	0.1 (0.01)	0.2 (0.01)	0.2 (0.01)	0.1 (0.01)	0.1 (0.01)	0.2 (0.01)
		>80	0.3 (0.01)	0.3 (0.01)	0.2 (0.01)	0.2 (0.01)	0.2 (0.01)	0.2 (0.01)	0.1 (0.01)
Oropharyngeal cancer									
	Female	15–64	0.3 (0.01)	0.2 (0.01)	0.2 (0.01)	0.2 (0.01)	0.2 (0.01)	0.2 (0.01)	0.1 (0.01)
		65–80	0.2 (0.01)	0.3 (0.01)	0.2 (0.01)	0.4 (0.01)	0.3 (0.01)	0.3 (0.01)	0.3 (0.01)
		>80	0.6 (0.01)	0.5 (0.01)	0.4 (0.01)	0.7 (0.01)	0.5 (0.01)	0.5 (0.01)	0.5 (0.01)
	Male	15–64	0.5 (0.01)	0.7 (0.01)	0.5 (0.01)	0.6 (0.01)	0.2 (0.01)	0.4 (0.01)	0.3 (0.01)
		65–80	1.0 (0.01)	1.1 (0.02)	1.0 (0.01)	0.9 (0.01)	0.6 (0.01)	1.0 (0.01)	0.9 (0.01)
		>80	1.3 (0.02)	0.9 (0.01)	0.7 (0.01)	1.2 (0.03)	0.8 (0.01)	1.4 (0.03)	1.6 (0.04)

## Data Availability

Data are available upon request due to privacy and ethical restrictions.
